# The effects of a pretreatment educational group programme on mental health treatment outcomes: a randomized controlled trial

**DOI:** 10.1186/s12913-018-3466-2

**Published:** 2018-08-29

**Authors:** John Morten Koksvik, Olav Morten Linaker, Rolf Wilhelm Gråwe, Johan Håkon Bjørngaard, Mariela Loreto Lara-Cabrera

**Affiliations:** 1 Department of Mental Health, Faculty of Medicine and Health Sciences, NTNU – Norwegian University of Science and Technology, Pb 8905 MTFS, 7491 Trondheim, Norway; 2 Tiller Community Mental Health Center, Division of Mental Health Care, St. Olavs University Hospital, Trondheim, Norway; 3 Department of Research and Development, Division of Mental Health Care, St. Olavs University Hospital, Trondheim, Norway; 4 Department of Public Health and Nursing, Faculty of Medicine and Health Sciences, NTNU – Norwegian University of Science and Technology, Trondheim, Norway; 5 Forensic Department and Research Center Brøset, St. Olavs University Hospital, Trondheim, Norway

**Keywords:** Patient education, Mental health, Patient dropout, Community mental health centers, Peer group, Randomised controlled trial, Self-management

## Abstract

**Background:**

Patients dropping out of mental health treatment is considered a widespread and significant obstacle to providing effective treatment, thus reducing the probability of patients achieving the desired improvement. Here, relative to ordinary treatment, we investigate the effects of providing an educational group programme before mental health treatment on mental health symptomatology and the risk of patients dropping out or prematurely discontinuing treatment.

**Methods:**

A randomized controlled trial in which adults referred to a community mental health center were randomized to either a Control Group (*n* = 46) or a pretreatment educational programme followed by treatment as usual (Intervention Group, *n* = 45). The primary outcome was self-reported mental health symptomatology assessed with BASIS-32. Data were analyzed by multilevel linear regression and Cox’s regression.

**Results:**

We recruited 93 patients during a 26-month period. Assessments were performed before (0 month, baseline) and after the intervention (1 month, before treatment initiation), and after 4 and 12 months. The net difference in BASIS-32 score between 0 and 1-month was − 0.27 (95% confidence interval CI] -0.45 to − 0.09) in favor of the intervention group. Although both groups had a significant and continuous decline in psychopathology during the treatment (from 1 month and throughout the 4- and 12-month follow-up assessments), the group difference detected before treatment (between 0 and 1 month) persisted throughout the study. Premature treatment discontinuation was partially prevented. The dropout risk was 74% lower in the Intervention Group than in the Control Group (hazard ratio 0.26, 95% CI = 0.07–0.93).

**Conclusions:**

A brief educational intervention provided before mental health treatment seems to have an immediate and long-lasting effect on psychopathology, supplementary to traditional treatment. Such an intervention might also have a promising effect on reducing treatment dropout.

**Trial registration:**

NCT00967265, clinicaltrials.gov. Registered August 27, 2009, retrospectively registered.

## Background

Successful mental health treatment requires active patient participation [1–3], and pretreatment educational group programmes might be a key strategy for achieving this goal. Pretreatment education provides a framework for preparation for therapy that aims to educate patients about the nature of, and rationale for treatment [4]. The key principles of pretreatment educational programmes are role induction and experiential pretraining [5]. Role induction involves providing the rationale for therapy and information about the roles of the patients and therapists, whereas experiential pretraining, which is typically conducted in a group setting, demonstrates how the therapy works. Pretreatment education might involve issues such as information about assessments, duration, and possible aims of therapy, as well as expectations, rights, and responsibilities of both the patient and the therapist in psychotherapy [4]. Previous research investigating pretreatment interventions has focused on clarifying therapy expectations [6–8], improving alliance [9, 10], mental health knowledge, mental health attitudes and barriers to treatment [11–13], patient awareness concerning the therapeutic process [14], and increasing knowledge about group psychotherapy [15, 16].

Research indicates that pretreatment educational interventions delivered before psychotherapy enhance the ability of patients to remain in treatment [5, 17], increase patient attendance [16, 18], and help patients develop behavioral skills [8, 15, 19, 20]. Recent studies suggest that pretreatment educational interventions administrated in co-operation with peers have a positive effect on patients’ knowledge about treatment preferences [21] and might lead to improved patient activation during the first months of treatment [22]. The possible supplementary effect of such interventions on mental health symptomatology has been questioned, but some studies have shown promising results [5, 22–24]. Although educational interventions might be particularly effective to support patients becoming actively engaged in their treatment, only a few studies have investigated the long-term effects of patient education on adherence or premature treatment discontinuation in mental health treatment. A systematic review by Greene et al. [17] included 11 studies on interventions to increase retention in mental health services, but only one study by Scott et al. [25] examined follow-up data after 6 months of treatment. Greene et al. [17] found, however, that comprehensive interventions targeting patient knowledge, mental health attitudes, and barriers related to treatment, have the potential of retaining patients in treatment until they achieve their treatment goals.

Retaining patients in mental health treatment is considered to be necessary to provide effective treatment. Research shows that a substantial number of outpatients receiving psychotherapy decide to end their treatment before they achieve the desired outcome [26–29]. A lack of improvement and dissatisfaction with therapy are frequently reported to be involved in patients’ decisions to leave treatment early [30]. Premature treatment discontinuation, also referred to as dropping out of therapy, is associated with worsened outcome [4, 27, 31–33], considerable economic costs [34, 35], and impaired service efficiency [36, 37].

At a Community Mental Health Center in central Norway, health personnel in cooperation with patient representatives and peer educators developed an educational programme for patients waiting for treatment. The main aim of the educational programme was to provide information to the patients about mental health, mental health treatment, self-management, and addressing the importance of actively engaging in their upcoming treatment. Although it has been demonstrated that this program increases mental health knowledge in the short-term [22], we do not know its long-term effects on psychopathology and treatment retention. Failure to realize that mental health treatment frequently takes a considerable length of time [38] might be one reason for patients dropping out of treatment. Thus, making patients aware, at an early stage of the process, that therapy is likely to take time and requires much active involvement, might prevent premature treatment termination [39].

Here we investigated the effects of the educational programme on mental health symptomatology (primary outcome) after the intervention, 1 month after study inclusion, and during treatment (4 and 12 months after study inclusion). We also investigated the risk of dropping out (secondary outcome) during the study period.

## Methods

### Study design and randomization

This was a randomized clinical controlled trial, where patients were allocated to either the Intervention Group (IG), receiving a pretreatment educational group and treatment as usual, or the Control Group (CG), receiving only the usual treatment. The collection of data began in June 2009 and was completed in August 2013.

### Ethics

The study was conducted in accordance with the Helsinki Declaration, and was approved by the Regional Committee for Medical and Health Research Ethics in Central Norway (no. 4.2009.77). Patients did not receive payment for participation. The trial was registered in ClinicalTrials.gov (trial no. NCT00967265).

### Participants

The study was conducted at a Community Mental Health Center (CMHC) at St. Olavs University Hospital, with a catchment area covering urban and rural areas with a population of 90,000. The Community Mental Health Centers constitute the major part of the public mental health care in Norway. These centers have responsibility for the specialized mental health services for the population in their respective catchment areas, providing individual or group outpatient treatments, ambulatory, and residential services. Patients receiving outpatient treatment pay a fee up to a certain amount and are then entitled to an exemption card. Participants in this study were patients who had been referred for outpatient treatment and were waiting for treatment at one of the four CMHC outpatient units at St. Olavs University Hospital. Patients were eligible for inclusion in the trial if they were at least 18-years-old. Additional inclusion criteria were being able to understand the Norwegian language and had given informed consent to participate in the study. Patients were excluded from participation if they had significant language or comprehension difficulties, severe dyslexia or cognitive impairment.

### Recruitment and procedure

Potential participants were identified by the intake team at the clinics, and they were subsequently sent a letter that informed them about the study and invited them to participate. Five days after the invitation was sent, potential participants were called by an employee at the CMHC informing them about the study. Patients accepting the invitation were given an inclusion appointment and evaluation. Participants who met the inclusion criteria and gave written informed consent were asked to complete the baseline questionnaires.

### Assignment

The randomization was done after the baseline data collection, using an Internet-based computer program provided independently by the Research Trial Service Centre at the Norwegian University of Science and Technology. Researchers were not involved in the randomization process. A block randomization procedure without stratification was used, and the researchers were blind to the size of the blocks. Participants were randomized 1:1 to either the educational programme (the IG) or to the CG. The patients were informed of their group allocation immediately after the randomization.

### Blinding

This was an open study, and the patients and the persons administrating the educational interventions were not blinded to group allocation. Several strategies were nevertheless used to achieve masked ratings: the researchers were not involved in the randomization process, and the research assistant (registering treatment sessions and duration from the hospital data registry) and the researcher analyzing the data (JHB) were blinded to group allocation. Information on treatment duration, attendance to treatment sessions, and discontinuation of treatment were obtained from the hospital data registry by a blinded research assistant.

### The intervention and the control group

The objective of the intervention was to improve patient participation, patient activation, and self-management in mental health treatment by providing information and knowledge to the patients, with emphasis on the importance of actively engaging in their treatment process and their mental health situation. The intervention was developed in cooperation with the health personnel and peers with experience as user representatives in mental health services. The programme content was based on the literature on engagement interventions, user involvement, and self-management [5, 40–42], as well as the Norwegian health legislation (Specialist Health Services Acts and Patient Rights Acts) and the results from collaborative meetings with health personnel (n = 5) and user representatives (n = 4). Five patient participation elements were incorporated in the intervention: (a) patient knowledge about how the treatment works and their roles as active participants in the treatment process; (b) explicit encouragement of participants to actively contribute in the interventions by asking questions and sharing personal experiences and values; (c) recognition of the patients responsibility to actively take part in the treatment decision-making processes; (d) different possible treatment options - the possibility of changing therapist and self-management options; and (e) time and length of treatment [43].

The teaching in the educational programme was based on a combination of the shared expertise of health professionals, peer educators, and user representatives, as well as the philosophy of informed decision making, self-determination, shared responsibility, and self-management [40–42]. Before each educational group session, four health professionals and one user representative held a short meeting to review the program and planned practical aspects, such as small-group participation.

The intervention consisted of two 4-h educational group sessions, followed by an individual planning session (lasting 45 min with a clinical therapist within a week after the group sessions) and treatment as usual. The purpose of the individual planning session was to identify the patients’ specific problems and their mental health status and to discuss available treatment options. Up to 15 patients could participate in each group session.

The educational methods were lectures and small-group discussions co-facilitated by health professionals and patient representatives, in which the treatment possibilities and the self-management strategies [44] were discussed. Breaks were scheduled to give participants time to interact. During the breaks, self-help literature and leaflets from patient organizations were displayed. All participants received a folder of leaflets from government agencies about mental health disorders and treatment possibilities.

Before the study implementation and randomization, the user representatives functioning as peer educators received two training sessions concerning patient participation and to enhance their pedagogic skills. The health personnel did not receive any formal pedagogic training in advance.

Participants allocated to the CG received ‘treatment as usual’, in accordance with a standard treatment at the CMHC (without receiving any education before treatment). The CG also received standard written information about treatment possibilities and patients’ rights at the inclusion appointment.

### Outcome measurements

The baseline assessments were completed at the CMHC at the time of inclusion (before randomization). The primary outcome was collected at baseline and 1, 4, and 12 months follow-up. The respondents who did not return their questionnaires within a week were contacted by the research assistant, encouraging them to convey their answers. The follow-up questionnaires were mailed again up to two times if the participants still did not answer.

### Primary outcome

The primary pre-defined outcome was mental health symptomatology measured by the Behaviour and Symptom Identification Scale (BASIS-32) [45]. The BASIS-32 is a 32-item self-report questionnaire measuring the degree of difficulty patients have experienced regarding major areas of life functioning during the preceding week. The items range from 0 (no difficulty) to 4 (extreme difficulty). These 32 items assess five subscales: relations to self and others (seven items); daily living and role functioning (seven items); depression and anxiety (six items); impulsive and addictive behavior (six items); and psychosis (four items) [46]. Cronbach’s *α* coefficient was 0.91 in this study.

### Secondary outcome

The secondary outcome was the risk of patients dropping out of treatment during the study period. Treatment dropout, also referred to as premature treatment discontinuation, is defined as when a patient unilaterally decides to discontinue treatment without the therapist’s approval. This implies that the therapist is considering the patient to be in need of further treatment but, nevertheless, the patient fails to attend or does not show up for further treatment sessions. The patients that do not show up to a planned session, but are considered by their therapist to be in need of further treatment, are mailed a reminder letter encouraging them to attend to one or more sessions. If a patient does not respond to this reminder letter or does not attend further sessions, the patient is defined as a dropout in this study. If the patient is considered to have prematurely discontinued the treatment, the time of dropout is defined as the last attended treatment session.

The risk of premature treatment discontinuation was calculated based on the number of participants dropping out and the treatment duration in both the intervention and control conditions. Treatment duration is defined as the time from when the participant was included in the study to the time of treatment discontinuation. If the patient was still in treatment at the end of the study, the treatment duration was considered 12 months. If the patient discontinued the treatment during the first 12 months, the time of the last attended treatment session was registered by the therapist in the hospital data registry.

### Sample size

The sample size for the long-term effect after 1 year was calculated on the basis of the primary outcome measure BASIS-32. A sample size of 32 participants per group was estimated to be necessary to detect group differences, with a power of 0.8 and a 0.05 alpha. To allow for up to 30% withdrawals after randomization, we aimed for a total of 46 patients in each group.

### Data analysis

The estimation of the intervention effects was performed according to the intention to treat principle, using available cases. Baseline analyses were done with IBM Corp. SPSS, version 22.0 [47]. Demographic and clinical characteristics at baseline were analyzed using a two-tailed paired t-test or the chi-square test.

The primary outcome was analyzed with STATA (Stata Corp., College Station, TX), using a multilevel linear regression model with random slopes, that uses all the information available. This model is less susceptible to bias under the assumption of missing at random [48, 49].

A survival Cox regression model was used to analyze the time (duration) from inclusion in the study to the time of treatment discontinuation. The participants were followed from the start of treatment for 1 year or until dropout of treatment, whichever occurred first. The proportional hazard assumption was assessed on the basis of Schoenfeld residuals (independence between residuals and time), and there was no evidence of non-proportionality. Precision was measured with 95% confidence intervals (CIs).

## Results

### Recruitment and study attrition

**Fig. 1:**
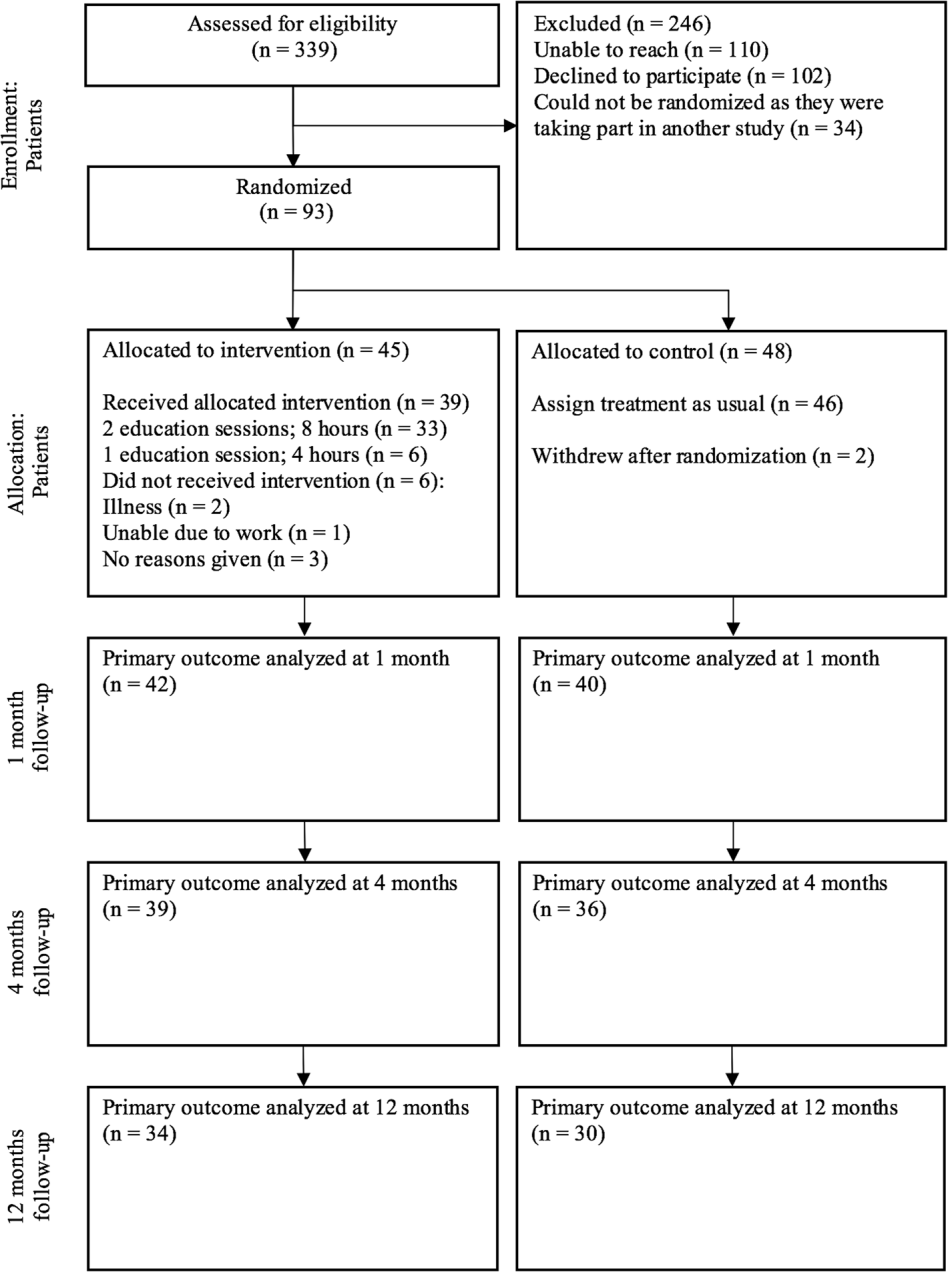
Recruitment and attrition according to CONSORT guidelines

Recruitment and attrition according to the CONSORT guidelines [50] are presented in Fig. 1. Letters of invitation were sent to 339 patients of whom 110 (32.4%) were unable to be reached. A total of 127 (37.5%) responded positively to the invitation, of whom 93 agreed to participate in the trial and were randomly assigned to the IG (*n* = 45) or CG (*n* = 48). Two participants who were randomized to the CG withdrew their consent after randomization and were not included in the analyses.

### Baseline characteristics

The IG and the CG were similar at baseline with respect to all demographic and clinical characteristics (Table 1).

**Table 1:** Descriptive demographic characteristics at baseline. Values are mean, standard deviation (SD) or number (%)

	IG (*n* = 45)	CG (*n* = 46)
Female, *n* (%)	29 (64)	35 (76)
Ethnicity (Norwegian)	43 (95.6%)	44 (95.7)
Mean age, y (SD)	38.36 (13.3)	37.09 (12.8)
Higher education, *n* (%)	11 (24%)	9 (19.6%)
Living with someone, *n* (%)	32 (54.2%)	27 (45.8%)
Employed, *n* (%)	9 (20%)	11 (23.9%)
Married, *n* (%)	27 (60%)	18 (40%)
Mean BASIS-32^a^ (SD)	1.46 (0.7)	1.37 (0.5)

^a^Patients mental health status was assessed by the BASIS-32. Scores can range from 0 to 4

### Feasibility of the intervention

The educational intervention was held twice per year. Three user representatives who were peer-educators and eight health professionals (one psychiatrist; two clinical psychologists; one social worker; one physiotherapist; and three psychiatric nurses) co-led the educational sessions. The three peer-educators and six health professionals (two clinical psychologists; one physiotherapist; and three psychiatric nurses) took part in each small-group session.

Thirty-nine of 45 participants (87%) took part in the educational intervention, 33 attended both sessions, and six attended one session. Six participants did not participate due to no-show or illness (Fig. 1). The individual planning session after the educational engagement session was held within 1 week after the intervention for 38 of 45 participants (84%). No one in the CG received the educational intervention.

### Follow-up and attrition

Follow-up questionnaires were completed at 1 month by 82 (88.2%) participants (IG, *n* = 42; CG, *n* = 40), by 75 (80.6%) participants (IG, *n* = 39; CG, *n* = 36) at 4 months, and by 64 (68.8%) participants (IG, *n* = 34; CG, *n* = 30) 12 months after baseline (Fig. 1). Response during follow-up was assessed with a random intercept logistic regression model. We found lower response during follow-up for the CG compared to the IG (odds ratio 0.56, 95% CI = 0.30–1.06).

### Primary outcome: Mental health symptomatology

**Fig. 2:**
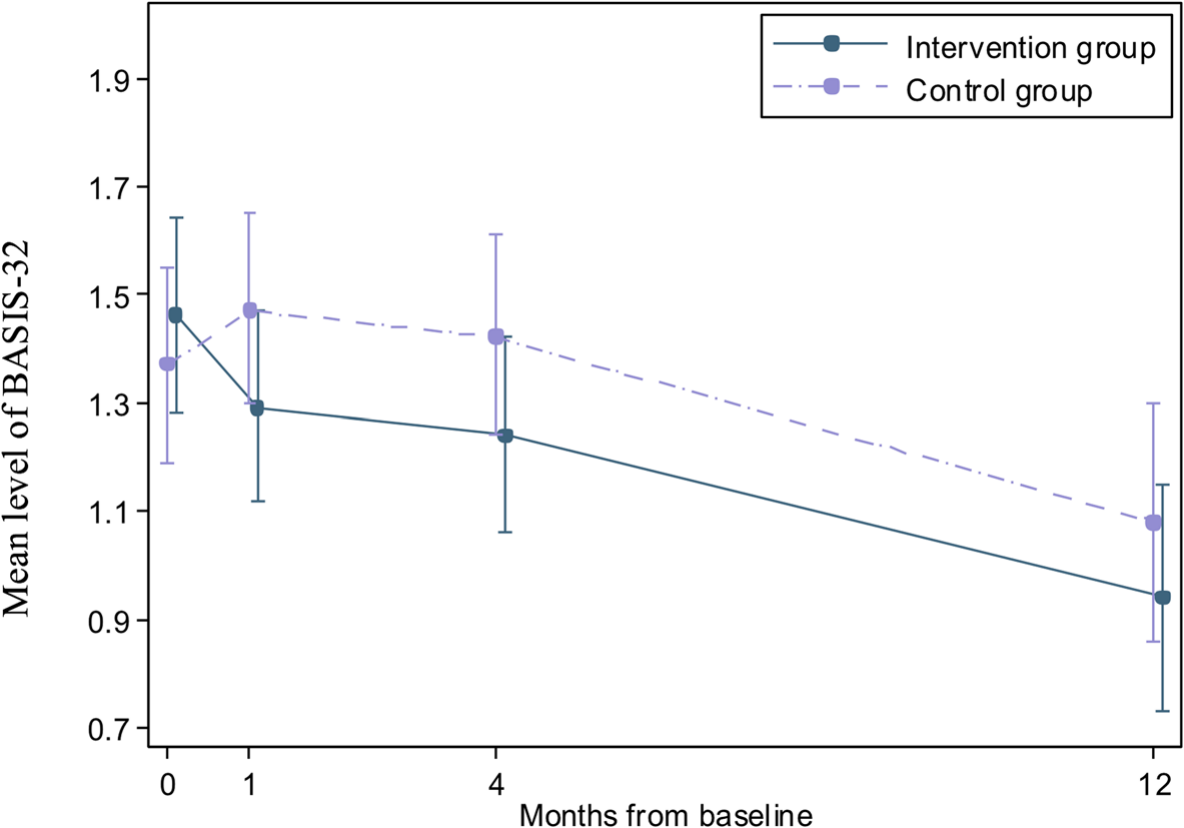
The estimated level of BASIS-32 at baseline (0), 1 month, 4 months, and 12 months follow-up. 95% confidence intervals (vertical lines)

As expected, at baseline there were no substantial group differences regarding mental health symptomatology (Fig. 2). At 1-month follow-up, the IG had an immediate reduction in symptom pressure with a − 0.16 scale points reduction (95% CI = − 0.29 to − 0.03). The CG increased their symptom pressure by 0.11 scale points at 1-month follow-up (95% CI = − 0.02–0.24). The net difference in BASIS score between 0- to 1-month follow-up was − 0.27 (95% CI = − 0.45 to − 0.09) in favor of the IG. Estimations are based on the results from a linear mixed model.

Although both groups showed a continuous decline in mental health symptomatology from 1 month and throughout the 4 and 12 months follow-up assessments, the group difference that occurred at 1 month persisted throughout the study. The net difference in BASIS score between 0 to 4 months and 0 to 12 months follow-up was − 0.27 (95% CI = − 0.50, − 0.05) and − 0.23 (95% CI = − 0.52 to − 0.06) respectively, in favour of the IG.

The IG showed a change in mental health symptomatology of − 0.35 (95% CI = − 0.52 to − 0.18) from 1 to 12 months follow-up, while the equivalent change within the CG was − 0.39 (95% CI = − 0.57 to − 0.21).

### Secondary outcome: Treatment dropout

**Fig. 3:**
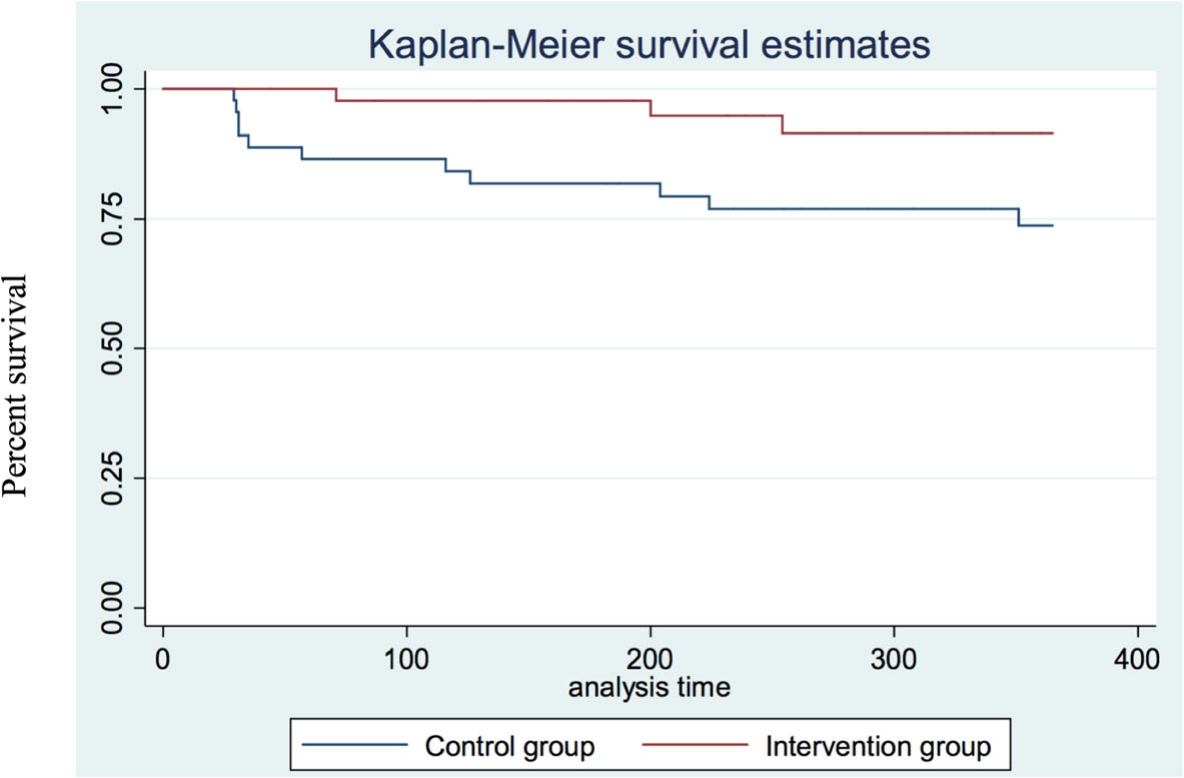
Kaplan-Meier survival curve showing the dropout rates for the IG and the CG

During the 12 months follow-up study period after baseline, a total of 14 (15.4%) participants prematurely terminated treatment, 3 of 45 respondents dropped out in the IG, and 11 of 46 dropped out in the CG. All of the respondents showed up to at least one treatment session before they either completed or dropped out of treatment. A Cox’s regression model showed that the dropout risk was 74% lower in the IG compared with the CG (hazard ratio 0.26, 95% CI = 0.07–0.93). The difference in treatment dropout rate between the IG and the CG during the 12 months study period is illustrated in Fig. 3, as a Kaplan-Meier survival curve.

## Discussion

The main finding of this randomized controlled trial was that, based on mental health symptomatology, providing an educational group programme before outpatient mental health treatment shows an immediate and long-lasting supplementary effect to the mental health treatment. The group difference in symptom reduction occurred after the intervention (and before treatment initiation). Moreover, this initial reduced symptomatology effect lasted throughout the 12 months of treatment. Contrarily, the CG reported a small deterioration in general psychopathology from baseline to treatment initiation, and the equivalent improvement in this group (compared to the IG) did not take place until after 3 months of treatment (at 4 months follow-up). Another finding with respect to the secondary outcome was that patients who did not participate in the pretreatment educational group programme experienced a 74% higher risk of prematurely discontinuing their following treatment compared to those participating in the programme before treatment. This noteworthy finding suggests that educational interventions provided before mental health treatment have an immediate and supplementary effect, in addition to the following treatment, on mental health symptomatology, and might be of considerable importance for providing a more long-lasting preventive effect on treatment dropout. Our findings also suggest that brief pretreatment educational interventions targeting mental health knowledge, providing information about mental health treatment, and addressing the importance of user involvement, might increase treatment retention and help patients remain in treatment.

The difference between the IG and CG regarding the reduction in mental health symptomatology are in contrast with a study of Lara-Cabrera et al. [22], who found that a similar intervention, only shorter in time, was not associated with symptom reduction. This difference might be a result of the dose of the intervention or the higher statistical power in this study. There was a non-significant difference in the study of Lara et al. favoring the experimental group who received the educational intervention. Our findings extend the findings of Green et al. [24], who also found significant reductions in mental health symptoms and improved functioning due to a similar intervention. All studies, including our study, used the group format with both professional mental health personnel, trained peer educators, and patient representatives to promote engagement.

The reduced risk of prematurely discontinuing treatment among patients receiving the treatment preparation intervention is in accordance with previous research. In their systematic review, Greene et al. [17] found that comprehensive interventions targeting patient knowledge, mental health attitudes, and barriers related to treatment, such as the intervention in this study, show promising results for retaining patients in mental health services. Helping patients to remain in mental health treatment also increases the possibility that they are improving and achieving their treatment goals. The effect sizes in the studies of such interventions vary to a great deal [17], but the presence of even small differences might be of clinical importance [51].

However, previous research findings are mainly based on follow-up data no longer than 6 months of treatment. The results of our study show an important consistent and persistent pattern, suggesting that these findings are of a persisting supplementary value to ordinary mental health treatment, thus giving the patients a better chance of achieving positive results from treatment. However, without having assessed the cost-effectiveness of the intervention, it is not controversial to assume that this kind of interventions is rather inexpensive and easy to administrate. Nevertheless, we believe that the use of health professionals, peer educators, and patient representatives to provide a pretreatment educational program is an innovative model that facilitates user involvement in mental health services.

### Strengths and limitations

The use of a randomized controlled trial (RCT) design with wide inclusion criteria in a standard clinical setting at a CMHC make these effectiveness findings highly relevant for good clinical practice in mental health treatment programmes. Adding brief educational groups to standard treatment regimens might easily fit into the everyday running of a CMHC. Moreover, the educational programme requires a limited use of hospital resources and takes little extra time. Such initiatives are also in accordance with recommendations aiming to improve attendance in mental health services [35]. The use of patient representatives and peer educators as co-leaders of the educational programme, as done in this study, implies an emphasis on experiential knowledge that is complementary to that of healthcare professionals. As previous research has stated, there is little empirical evidence of change resulting from service user involvement [52–54]. Thus, this study exemplifies a potential organizational structuring, where service user involvement is integrated into mental health care, in a way that seems to add an important supplement to traditional mental health treatment.

A strength of this study was the inclusion criteria, which were wide and general and did not exclude important patient groups, but some caution should be applied regarding the generalizability of the study. A relatively high number of patients who were asked to participate did not respond and were, therefore, excluded from the study. One-third of the patients invited could not be reached, they did not respond to either letters or phone calls. One-third of the patients declined participation. We do not know the reasons why so many eligible patients decided not to participate in the study, but the group format of the intervention, confidentiality uncertainty, or the possible stigma related to being a psychiatric patient may have been reasons for this. One further reason may be that the educational intervention was held during the day and, for many patients, work hours, making it difficult to participate. As treatment fees are rather limited for attending outpatient treatment in Norway, we do not believe that economical cost would account for the high number of patients deciding not to participate in the study. Nevertheless, the participation rate is comparable to other RCTs also performed in a natural setting such as routine mental health care and drug addiction services.

Other study limitations must also be considered. This work was based on a small sample, calculated on the basis of the power required to demonstrate differences in the effect measure (BASIS-32). It would, however, have been preferable to also have powered the study on the dropout measure. The questions regarding the clinical significance of the reductions in overall mental health symptomatology, the wide confidence intervals, and the considerable number of eligible patients who declined to participate in the study, encourage caution with respect to the generalization of the findings. Because this study was conducted at outpatient clinics at a Community Mental Health Centre, the results might not be representative of other mental health settings. Another limitation of this study is that neither patients nor therapists were blind to allocated groups.

Future studies must carefully consider the methodological challenges and limitations outlined here. An important contribution of future research would be to power trials to make it possible to examine predictors of positive outcomes, such as diagnosis, demographic factors, treatment factors etc. Further studies involving a higher number of participants will also contribute to minimizing possible bias caused by variations in therapist qualifications, experience, and degree of evidence-based treatment provided.

There is some evidence showing that intervention strategies targeting knowledge, attitudes, and barriers to mental health treatment are significantly associated with reduced dropout [17]. Such interventions have typically been added to the standard treatment and have differed considerably with respect to modes of delivery (e.g., phone, group, individual), duration, and frequency. To optimize the effects of such interventions on psychopathology and retention, we believe that future studies should focus on developing strategies to integrate such interventions into standard clinical pathways and to assess the cost-effectiveness of such educational interventions.

### Conclusions

This study represents an important first step in the evaluation of educational group intervention co-delivered by peer educators, indicating that providing a brief and comprehensive educational group program for outpatients might result in an initial reduction in psychopathology. The pretreatment educational intervention has a valuable supplementary treatment effect on mental health and likely also leads to an important reduced risk of dropping out of treatment. As such, this finding suggests that the participation in pretreatment educational groups encourages patients to remain in treatment and also helps to ensure that they experience improvement from attending treatment sessions as planned. These findings are particularly relevant for outpatient treatment where patients need support to become actively involved in their treatment. We believe that the use of health professionals, peer educators, and patient representatives in the intervention is likely to have a positive effect on how the participants experience the educational program.

To summarize, the findings from our study provide initial support for the clinical value of pretreatment interventions. To draw more certain conclusions on the effects of pretreatment programmes on dropout, future randomized controlled studies should include active control groups and be powered to assess this outcome. Research on the relative effectiveness of how to provide educational programmes could help determine the most cost-effective allocation of resources. Efforts to strengthen the evidence of pretreatment educational group interventions require robust studies, allowing the identification of the mechanisms of change in this intervention as well as cost-benefit studies.

## Abbreviations

BASIS-32: Behavior and Symptom Identification Scale, 32 items
CG: Control Group
CI: Confidence Interval
CMHC: Community Mental Health Center
CONSORT: Consolidated Standards of Reporting Trials
IG: Intervention Group
RCT: Randomized Controlled Trial
SD: Standard Deviation

## References

[1] Gruman J, Rovner MH, French ME, Jeffress D, Sofaer S, Shaller D, Prager DJ (2010). From patient education to patient engagement: implications for the field of patient education. Patient Educ Couns.

[2] Coulter A (2012). Patient engagement—what works?. The Journal of Ambulatory Care Management.

[3] Simmons LA, Wolever RQ, Bechard EM, Snyderman R (2014). Patient engagement as a risk factor in personalized health care: a systematic review of the literature on chronic disease. Genome Medicine.

[4] Oldham M, Kellett S, Miles E, Sheeran P (2012). Interventions to increase attendance at psychotherapy: a meta-analysis of randomized controlled trials. J Consult Clin Psychol.

[5] Walitzer KS, Dermen KH, Connors GJ (1999). Strategies for preparing clients for treatment a review. Behav Modif.

[6] Lambert RG, Lambert MJ (1984). The effects of role preparation for psychotherapy on immigrant clients seeking mental health services in Hawaii. Journal of community psychology.

[7] Deane FP, Spicer J, Leathem J (1992). Effects of videotaped preparatory information on expectations, anxiety, and psychotherapy outcome. J Consult Clin Psychol.

[8] Fende Guajardo JM, Anderson T (2007). An investigation of psychoeducational interventions about therapy. Psychother Res.

[9] Jónsson H, Hougaard E, Bennedsen B (2011). Randomized comparative study of group versus individual cognitive behavioural therapy for obsessive compulsive disorder. Acta Psychiatr Scand.

[10] Strassle CG, Borckardt JJ, Handler L, Nash M (2011). Video-tape role induction for psychotherapy: moving forward. Psychotherapy.

[11] Grote NK, Zuckoff A, Swartz H, Bledsoe SE, Geibel S (2007). Engaging women who are depressed and economically disadvantaged in mental health treatment. Soc Work.

[12] Alvidrez J, Areán PA, Stewart AL. Psychoeducation to Increase Psychotherapy Entry for Older African Americans. *The American Journal of Geriatric Psychiatry*. 2005;13(7):554–61. 10.1176/appi.ajgp.13.7.554 16009731

[13] Sirey JA, Bruce ML, Alexopoulos GS (2005). The treatment initiation program: an intervention to improve depression outcomes in older adults. Am J Psychiatr.

[14] Anderson T, Strupp HH (1996). The ecology of psychotherapy research. J Consult Clin Psychol.

[15] Latour D, Cappeliez P (1994). Pretherapy training for group cognitive therapy with depressed older adults. Canadian Journal on Aging.

[16] Garrison JE (1978). Written vs verbal preparation of patients for group psychotherapy. Psychotherapy: Theory, Research & Practice.

[17] Greene JA, Bina R, Gum AM (2016). Interventions to increase retention in mental health services: a systematic review. Psychiatr Serv.

[18] France DG, Dugo JM (1985). Pretherapy orientation as preparation for open psychotherapy groups. Psychother Theory Res Pract Train.

[19] Kivlighan DM, Corazzini JG, McGovern TV (1985). Pregroup training. Small Group Behav.

[20] Strupp HH, Bloxom AL (1973). Preparing lower-class patients for group psychotherapy: development and evaluation of a role-induction film. J Consult Clin Psychol.

[21] Lara-Cabrera ML, Gjerden M, Gråwe RW, Linaker OM, Steinsbekk A (2016). Short-term effects of a peer co-led educational programme delivered before mental health treatment: a randomised controlled trial. Patient Educ Couns.

[22] Lara-Cabrera ML, Salvesen Ø, Nesset MB, De las Cuevas C, Iversen VC, Gråwe RW (2016). The effect of a brief educational programme added to mental health treatment to improve patient activation: a randomized controlled trial in community mental health centres. Patient Educ Couns.

[23] Piper WE, Perrault EL (1989). Pretherapy preparation for group members. Int J Group Psychother.

[24] Green CA, Janoff SL, Yarborough BJH, Paulson RI (2013). The recovery group project: development of an intervention led jointly by peer and professional counselors. Psychiatr Serv.

[25] Scott J, Colom F, Popova E, Benabarre A, Cruz N, Valenti M, Goikolea JM, Sánchez-Moreno J, Asenjo MA, Vieta E (2009). Long-term mental health resource utilization and cost of care following group psychoeducation or unstructured group support for bipolar disorders: a cost-benefit analysis. The Journal of Clinical Psychiatry.

[26] Wierzbicki M, Pekarik G (1993). A meta-analysis of psychotherapy dropout. Prof Psychol Res Pract.

[27] Barrett MS, Chua W-J, Crits-Christoph P, Gibbons MB, Thompson D (2008). Early withdrawal from mental health treatment: Implications for psychotherapy practice. Psychotherapy: Theory, Research, Practice, Training.

[28] Wells JE, Oakley Browne M, Aguilar-Gaxiola S, Al-Hamzawi A, Alonso J, Angermeyer MC, Bouzan C, Bruffaerts R, Bunting B, Caldas-de-Almeida JM, et al. Drop out from out-patient mental healthcare in the World Health Organization’s world mental health survey initiative. Br J Psychiatry. 2013; 10.1192/bjp.bp.112.113134 23174514

[29] Swift JK, Greenberg RP (2012). Premature discontinuation in adult psychotherapy: a meta-analysis. J Consult Clin Psychol.

[30] Kegel AF, Flückiger C (2015). Predicting psychotherapy dropouts: a multilevel approach. Clinical psychology & psychotherapy.

[31] Orhon FS, Soykan A, Ulukol B (2007). Patient compliance to psychiatric interventions and course of postpartum mood disorders. The International Journal of Psychiatry in Medicine.

[32] Killaspy H, Banerjee S, King M, Lloyd M (2000). Prospective controlled study of psychiatric out-patient non-attendance characteristics and outcome. Br J Psychiatry.

[33] Pekarik G (1983). Follow-up adjustment of outpatient dropouts. Am J Orthopsychiatry.

[34] Klein EB, Stone WN, Hicks MW, Pritchard IL (2003). Understanding dropouts. J Ment Health Couns.

[35] Bech M (2005). The economics of non-attendance and the expected effect of charging a fine on non-attendees. Health policy.

[36] Joshi PK, Maisami M, Coyle JT (1986). Prospective study of intake procedures in a child psychiatry clinic. J Clin Psychiatry.

[37] Rusius CW (1995). Improving out-patient attendance using postal appointment reminders. Psychiatr Bull.

[38] Mueller M, Pekarik G (2000). Treatment duration prediction: Client accuracy and its relationship to dropout, outcome, and satisfaction. Psychotherapy: Theory, Research, Practice, Training.

[39] Swift JK, Callahan JL (2008). A delay discounting measure of great expectations and the effectiveness of psychotherapy. Prof Psychol Res Pract.

[40] Fraenkel L, McGraw S (2007). What are the essential elements to enable patient participation in medical decision making?. J Gen Intern Med.

[41] Holman H, Lorig K (2004). Patient self-management: a key to effectiveness and efficiency in care of chronic disease. Public Health Rep.

[42] Lawn S, Battersby MW, Pols RG, Lawrence J, Parry T, Urukalo M (2007). The mental health expert patient: findings from a pilot study of a generic chronic condition self-management programme for people with mental illness. Int J Soc Psychiatry.

[43] Fraenkel L, McGraw S (2007). Participation in medical decision making: the Patients’ perspective. Med Decis Mak.

[44] Lorig KR, Sobel DS, Ritter PL, Laurent D, Hobbs M (2001). Effect of a self-management program on patients with chronic disease. Effective clinical practice: ECP.

[45] Eisen SV, Dill DL, Grob MC (1994). Reliability and validity of a brief patient-report instrument for psychiatric outcome evaluation. Psychiatr Serv.

[46] Klinkenberg WD, Cho DW, Vieweg B (1998). Reliability and validity of the interview and self-report versions of the BASIS-32. Psychiatr Serv.

[47] Corp I (2013). IBM SPSS statistics for windows, version 22.0.

[48] Rabe-Hesketh S, Skrondal A (2008). Multilevel and longitudinal modeling using STATA: STATA press.

[49] Wood AM, White IR, Thompson SG (2004). Are missing outcome data adequately handled? A review of published randomized controlled trials in major medical journals. Clinical Trials.

[50] Boutron I, Altman DG, Moher D, Schulz KF, Ravaud P, for the CNPTG (2017). Consort statement for randomized trials of nonpharmacologic treatments: a 2017 update and a consort extension for nonpharmacologic trial abstracts. Ann Intern Med.

[51] Rosenthal R: Meta-analytic procedures for social research, vol. 6: Sage; 1991.

[52] Crawford MJ, Rutter D, Manley C, Weaver T, Bhui K, Fulop N, Tyrer P (2002). Systematic review of involving patients in the planning and development of health care. BMJ.

[53] Millar SL, Chambers M, Giles M (2016). Service user involvement in mental health care: an evolutionary concept analysis. Health Expect.

[54] Lathlean J, Burgess A, Coldham T, Gibson C, Herbert L, Levett-Jones T, Simons L, Tee S (2006). Experiences of service user and carer participation in health care education. Nurse Educ Today.

